# Novel Treatment Approach to Ascending Aorta Pseudoaneurysms: A Case Report

**Published:** 2017-07

**Authors:** Seyed Ebrahim Kassaian, Seyed Khalil Forouzannia, Ali Hosseinsabet, Tahereh Davarpasand

**Affiliations:** *Tehran Heart Center, Tehran University of Medical Sciences, Tehran, Iran.*

**Keywords:** *Aneurysm, false*, *Aorta*, *Septal occluder device*

## Abstract

The pseudoaneurysm of the aorta is rarely due to trauma and infection but usually is a late complication of previous surgical procedures like coronary artery bypass graft surgery. We describe a 65-year-old woman with the pseudoaneurysm of the ascending aorta due to coronary artery bypass graft surgery. It was revealed in coronary artery angiography after nonspecific symptoms. It was confirmed by multidetector computed tomographic angiography. The patient refused open cardiac surgery, so we decided to use an atrial septal defect occluder device in off-label way to seal the pseudoaneurysm orifice. In a hybrid operating room setting, the procedure was done successfully and patient’s hospitalization course was eventless. In multidetector computed tomographic angiography after 3 months, the device was in the appropriate position without endoleak and in the yearly visit the patient was asymptomatic and healthy.

## Introduction

The pseudoaneurysm of the aorta (PSA) is rarely due to trauma and infection but usually is a late complication of previous surgical procedures like coronary artery bypass graft surgery (CABG).^1^^, ^^2^ Standard treatment is surgical intervention; however, it is often associated with high morbidity and mortality, which can reach up to 30%, especially in patients with a history of previous cardiac surgery.^2^^, ^^3^ One of the endovascular options for the PSA is an off-label use of occluder devices.^1^^, ^^4^ Here we discuss a case of post-CABG pseudoaneurysm treated via transcatheter intervention using an atrial septal defect (ASD) occluder device.

## Case Report

**Figure 1:**
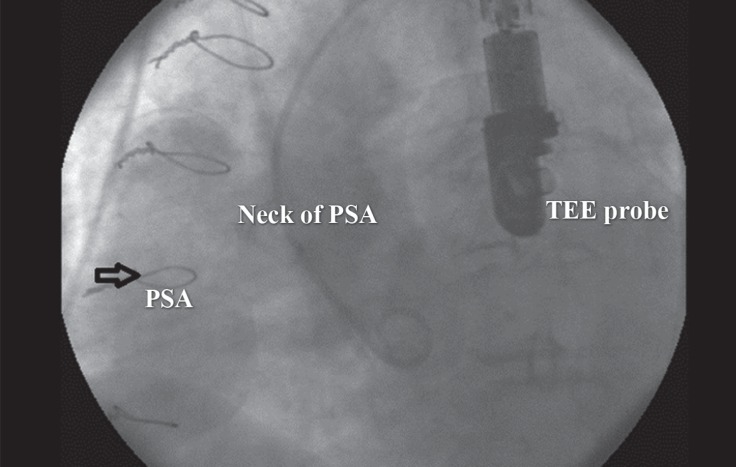
Preprocedural aortic catheterization in the lateral view shows a large pseudoaneurysm of the aorta (PSA) (arrow).

TEE, Transesophageal echocardiography; AA, Ascending aorta

**Figure 2:**
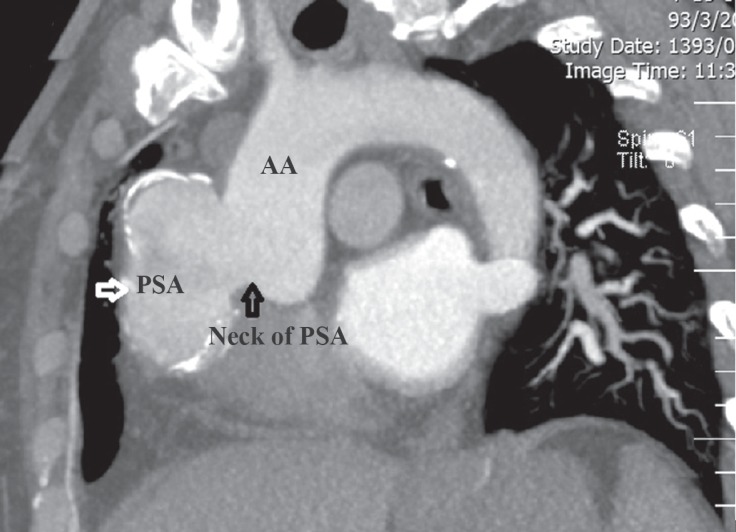
Multidetector computed tomographic angiography before the procedure shows the pseudoaneurysm of the ascending aorta (PSA).

**Figure 3:**
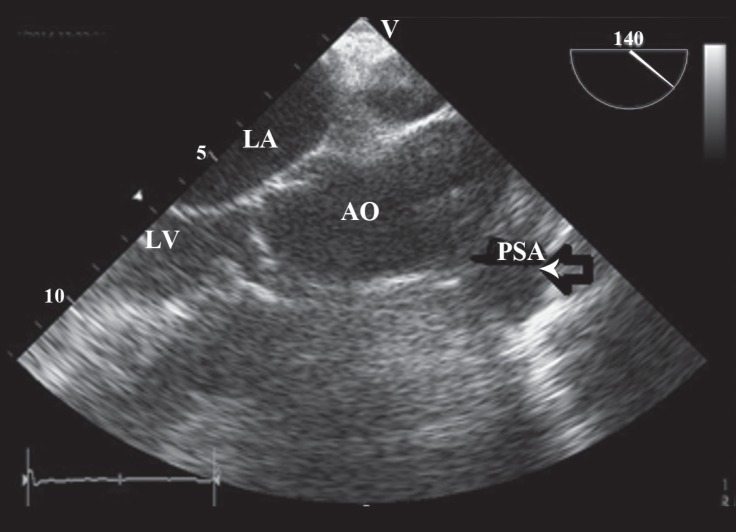
Preprocedural transesophageal echocardiography, parasternal long-axis view, shows the pseudoaneurysm of the aorta (arrow).

LV, Left ventricle; LA, Left atrium; AO, Aorta; PSA, Pseudoaneurysm of the aorta

A 65-year-old woman with controlled dyslipidemia and hypertension and a previous history of CABG 9 years previously was referred to our center because of the PSA. She had undergone coronary angiography due to symptoms which were suspected to be of ischemic origin in another hospital and during aortic root injection, the PSA was revealed. Coronary angiography showed that the left anterior descending artery and the right coronary artery were occluded at the proximal part; the left internal mammary on the left anterior descending artery was patent and the right coronary artery was filled retrogradely via the left coronary artery system. There was mild left ventricular systolic dysfunction. In addition, a large saccular aneurysm (25 × 55 mm) was detected in the ascending aorta (Figure 1). These findings were confirmed by multidetector computed tomographic (MDCT) angiography, which illustrated a large pseudoaneurysm in the tubular part of the ascending aorta (maximum aneurysm diameter and PSA neck were 60 mm and 24 mm, respectively) with a calcified wall and without evidence of leak or pericardial effusion (Figure 2). The diameters of the ascending aorta, arch, and descending aorta were 27, 20, and 17 mm, respectively, and the arch had 3 patent branches. In transesophageal echocardiography, the distance between the PSA and the aortic valve was 30 mm and the diameter of the oval-shaped orifice of the PSA was 20 × 29 mm. In addition, a thin layered clot was visible in the pseudoaneurysm cavity (Figure 3). Cardiac surgery consultation was done, but the patient refused open repair owing to the high risk of surgery. At this point, we arrived at the decision to use an ASD occluder device in off-label way so as to seal the PSA orifice. Written informed consent, after explanation, was obtained from the patient.

**Figure 4:**
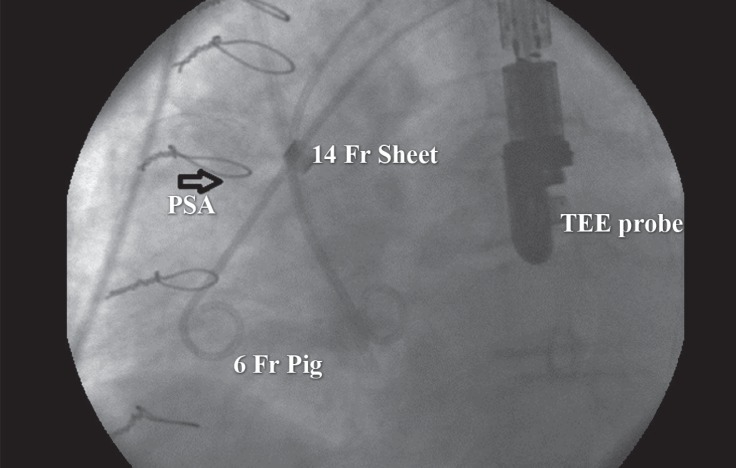
Preprocedural catheterization in the lateral view shows the entrance of a 6-F pigtail into the pseudoaneurysm of the aorta (PSA) (arrow).

TEE, Transesophageal echocardiography; Fr, French; Pig, Pigtail

**Figure 5:**
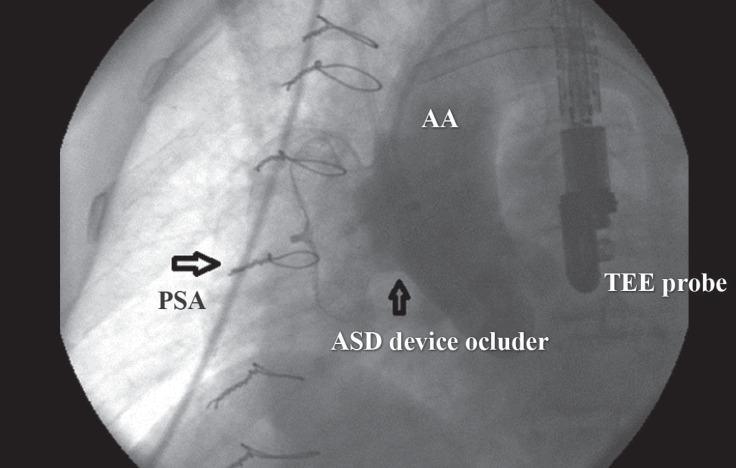
Postprocedural artic catheterization in the lateral view shows the atrial septal defect (ASD) device occluder in the appropriate site and no leakage.

TEE, Transesophageal echocardiography; AA, Ascending aorta; PSA, Pseudoaneurysm of the aorta

In a hybrid operating room setting, under general anesthesia and with transesophageal echocardiography guidance, after open arteriotomy, a 30-mm Amplatzer-like ASD occluder device (Cardi-O-Fix, Starway Medical Technology, Inc. Beijing, China) was delivered via the right femoral artery using a 14-F sheath and pigtail catheter. To avoid the risk of perforation or rupture of the pseudoaneurysm sac by direct guide wire contact, we entered the pigtail catheter into the PSA cavity, with an approach similar to the insertion of the catheter for the left atrial appendage closure device (Figure 4). Then, the sheath was introduced over the pigtail catheter through the PSA cavity. Finally, after the device was well-positioned in the orifice, aortic root angiography and transesophageal echocardiography showed no residual endoleak (Figure 5).

**Figure 6:**
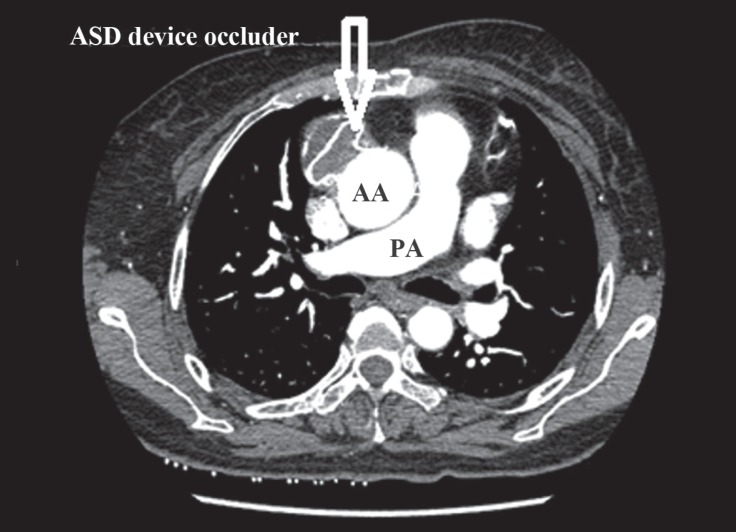
Postprocedural multidetector computed tomographic angiography shows the atrial septal defect (ASD) device occluder in the appropriate site and no leakage.

AA, Ascending aorta; PA, Pulmonary artery

The patient’s hospitalization course was eventless. Although dual antiplatelet therapy is not necessary for interventional procedures in the aorta considering the high flow pressure, our patient was low-risk for major bleeding and she was discharged healthy on Aspirin (80 mg/daily), off-label usage of Plavix (75 mg/daily for the first 3 months), Nitrocontin (2.6 mg/BD), metoprolol (25 mg/BD), atorvastatin (20 mg/daily), and ranitidine (150 mg/BD). At 3 months’ follow-up, MDCT angiography was repeated and it demonstrated that the device was in the appropriate position without endoleak (Figure 6). Once more, we visited the patient 1 year after the procedure and she had been asymptomatic and healthy since hospital discharge. The patient refused our request for repeat CT angiography, which can be deemed a limitation in our follow-up period. 

## Discussion

The PSA, albeit rarely in consequence of trauma and infection, is usually a late complication of previous surgical procedures such as CABG.^1^^, ^^2^ In most cases, the PSA is anteriorly located in the previous site of aortic cannulation.^4^ We inferred that our patient’s PSA was an iatrogenic complication after CABG at the site of a previous vein graft anastomosis of the right coronary artery which was located in the anterolateral wall of the ascending aorta.

Surgical intervention with direct aortic reconstruction constitutes standard treatment in patients with acceptable operative risk. Nonetheless, surgery is often associated with high morbidity and mortality, especially in patients with a history of previous surgery.^2^^, ^^4^ Given that the lethal complications of the PSA render intervention mandatory, percutaneous interventions have received a great deal of attention in recent years.^1^^, ^^4^

Given the risk of the rupture of the left internal mammary artery due to reoperation by the anterior surgical approach and the probability of pseudoaneurysm and aortic rupture as a result of aortic wall fragility, we sought a nonsurgical procedure. In lieu of reoperation, there are different percutaneous intervention methods for repairing the PSA in high-risk patients such as stent grafting, coil, and Glue.^3^^, ^^4^ Despite present controversies about different options, we opted for the ASD occluder device after discussion with our heart team members, consisting of a cardiovascular surgeon, an echocardiographist, and an interventional cardiologist.

In a recent study by Roselli et al.,^5^ stent grafting was used for 9 patients with an iatrogenic PSA and in the short- and long-term follow-up, surgical reintervention was performed for 2 patients due to stent migration and endoleak. The authors concluded that the limitations of stent grafting for the PSA included risk of coronary ostial coverage, device migration, endoleak, and absence of suitable devices. Overall, that study showed that endovascular therapy for these patients was feasible and life-saving. Elsewhere, Preventeza et al.^6^ performed endoluminal repair by stent grafting in 5 PSA patients and used the Amplatzer occluder device in 1 PSA patient and concluded that the latter procedure was associated with continuous endoleak in the early postoperative period. The authors, accordingly, covered the device with a thoracic Gore-TAG endograft (WL Gore and Associates Inc., Flagstaff, AZ, USA). Their findings suggested that stent grafting for the PSA was feasible but limited and technical expertise was essential; nevertheless, no recommendation was offered on the use of the Amplatzer device in these cases.

There are some case reports on the successful usage of the ASD device closure in patients with the PSA via different approaches, although this procedure can be associated with lethal morbidities such as device embolization.^3^^, ^^4^^, ^^7^ In a case series, Hussain et al.^8^ employed the Amplatzer device successfully to treat 6 patients with the PSA. (One patient had a recurrent pseudoaneurysm after previous occluder device exclusion.) Open surgery was necessary in 2 patients in their short-term follow-up. One of these patients had a history of previous surgical graft deployment for aortic dissection and the site of the PSA was at the junction of the aortic graft and the native aorta, in the proximal part of the aortic arch. The other patient had 2 large pseudoaneurysms and missed aortic dissection was found during device deployment. In contrast to the study by Hussain et al., in our patient the PSA site was in the ascending aorta and there was only 1 pseudoaneurysm and aortic dissection was ruled out. Obviously, patient selection is highly important in achieving a successful result. 

We demonstrated short-term success of the use of the ASD occluder device in the treatment of the PSA, although there are no known long-term follow-up data yet in comparison with open techniques. However, recently, an excellent mid-term 2-year follow-up result was reported by Stasek et al.^9^ after the percutaneous closure of a PSA using the Amplatzer ASD occluder. Also Successful percutaneous repair of a 40 × 27 mm ascending aorta pseudoaneurysm with an 8-mm Amplatzer Vascular Plug II was recently demonstrated by Garcı´a et al.^10^

As was in the case in our patient, MDCT angiography with electrocardiography gating can provide important diagnostic information in many cases of postoperative aortic root pseudoaneurysms.^9^ At 3 months’ follow-up, repeat MDCT showed that the device was in the appropriate position and there was no endoleak.

AA, Ascending aorta

## Conclusion

In light of our experience with the patient described here and the results of some previous studies, we would suggest that, despite the existing controversy, the use of the ASD occluder device for the treatment of the PSA might be a good choice for appropriately selected patients and could be considered an alternative method. Nonetheless, further experience and long-term follow-up are required.

## Notes:

***This paper should be cited as: ***Kassaian SE, Forouzannia SK, Hosseinsabet A, Davarpasand T. Novel Treatment Approach to Ascending Aorta Pseudoaneurysms: A Case Report. J Teh Univ Heart Ctr 2017;12(3):134-137.
